# Importance of characterising sleep breaks within the 24-h movement behaviour framework

**DOI:** 10.1186/s12966-021-01241-5

**Published:** 2022-01-06

**Authors:** Séverine Sabia, Manasa Shanta Yerramalla, Teresa Liu-Ambrose

**Affiliations:** 1 Université de Paris, Inserm U1153, CRESS, Epidemiology of Ageing and Neurodegenerative diseases, 10 avenue de Verdun, 75010 Paris, France; 2 Department of Epidemiology and Public Health, University College, London, UK; 3 Aging, Mobility, and Cognitive Neuroscience Laboratory, Department of Physical Therapy, University of British Columbia, Vancouver, British Columbia Canada; 4 Djavad Mowafaghian Centre for Brain Health, Vancouver Coastal Health Research Institute, Vancouver, British Columbia Canada; 5 Centre for Hip Health and Mobility, Vancouver Coastal Health Research Institute, Vancouver, British Columbia Canada

**Keywords:** Sleep, Physical activity, Sedentary time, Sleep breaks

## Abstract

Accelerometers measure the acceleration of the body part they are attached and allow to estimate time spent in activity levels (sedentary behaviour, light, and moderate-to-vigorous physical activity) and sleep over a 24-h period for several consecutive days. These advantages come with the challenges to analyse the large amount of data while integrating dimensions of both physical activity/sedentary behaviour and sleep domains. This commentary raises the questions of 1) how to classify sleep breaks (i.e. wake after sleep onset) during the night within the 24-h movement behaviour framework and 2) how to assess their impact on health while also accounting for night time sleep duration and time in sedentary behaviour and physical activity during the day. The authors advocate for future collaborations between researchers from the physical activity/sedentary behaviour and sleep research fields to ensure appropriate analysis and interpretation of the tremendous amount of data recorded by the newer generation accelerometers. This is the only way forward to provide meaningfully accurate evidence to inform future 24-h movement behaviour guidelines.

## Commentary

Physical activity, sedentary behaviour, and sleep are recognised behavioural factors critical for health. Technological advances have led to the use of wearables, such as accelerometers and gyroscopes, to capture these 24-h movement behaviours. These devices are increasingly being used in large-scale epidemiological studies, such as in the UK Biobank [1] or the National Health and Nutrition Examination Survey 2011–2014, [2] to assess movement behaviours across several consecutive days and nights. This is a considerable step forward as previous generation accelerometers to assess physical activity measurements usually were removed during night time. How to best utilise the vast amount of data on sedentary behaviours, physical activity, and sleep over a 24-h period is the challenge to be addressed for the coming years. This requires close collaborations between researchers from the field of physical activity and sleep, as well as from experts in analysis of accelerometer data. This commentary will describe the challenges related to analysis and interpretation for accelerometer-assessed data, as well as some thoughts for future directions.

Until recently, movement behaviour characteristics during waking time and night time were examined separately. On one hand, in the physical activity/sedentary behaviour field where accelerometers were worn mainly during waking hours, movement behaviours were categorised as sedentary behaviour, light, moderate, or vigorous physical activity. Once each time segment, i.e. epoch, was classified as a specific activity level, other characteristics such as time, frequency, and length of bouts for each activity level were further examined. On the other hand, in the sleep research field, each time segment within the time window from when the person goes to bed to when the person woke up to start the day, movement behaviours were classified as either awake or asleep states. From this data, multiple metrics to characterize sleep duration and its quality such as total sleep time, time in bed, sleep efficiency, sleep latency, wake after sleep onset were then derived.

The newer generation accelerometers now allow 24-h assessment of movement behaviours for several consecutive days. These advantages come with the need to harmonize terminology from both physical activity and sleep expertise fields. From the viewpoint of an expert in physical activity, breaks in sleep period are seen as sedentary behaviour if lying down while awake or light activity if ambulatory behaviours are undertaken. Whereas from a sleep experts’ standpoint, breaks in sleep period correspond to awake state during which the individuals try to fall asleep. From a behavioural perspective, while individuals can decide to be physically active or to sit during the day, the time awake during the night is not on purpose and thus can hardly be classified as being modifiable. In 2018, Gibbs & Kline [3] raised this issue and called for the differentiation of sedentary behaviour and sleep-related behaviour (defined as “waking behaviour where subject is attempting to fall asleep or return to sleep”) during the sleep period.

For health research purposes, it is necessary to differentiate sedentary behaviour during day time from when people try to fall asleep (sleep latency, wake after sleep onset) in order to provide their respective impact on health and study their different underlying physiological mechanisms. We recommend for the differentiation between these states, particularly for populations with high prevalence of sleep disturbances such older adults, individuals presenting obesity, those suffering from chronic pain or in clinical settings. Similarly, it is possible that light activities during the night such as walking in the corridor, having a cup of tea or a short snack that could be correlates of sleep disturbance might be deleterious for health, while light activities during the day are more likely to be beneficial. Considering movements during day time and those while awake during the sleep period as similar states is likely to bias findings on their role for health. In this context, it is important to remind the importance of sleep detection methods, as they have been shown to impact estimations of non-wear and sedentary time [4]. For example, it has been suggested that specific question on shuteye time (i.e., the time one attempts to fall asleep once in bed), instead of bedtime (i.e., the time a person goes to bed), is more useful as a complement to movement sensors to differentiate sedentary behaviour before sleep (such as reading or watching television in bed) from sleep latency (i.e., the amount of time taken to fall asleep after attempting to go to sleep) [5].

When analysing accelerometer-assessed data, it is essential to give consideration on how movements when awake during the sleep period are classified. In 2015, an analytical method was proposed to deal with the compositional nature of 24-h movement behaviour data [6]. This approach allows to account for the co-dependency of movement behaviours resulting from the fact that an increase in time spent in one behaviour – often classified as sedentary, light activity, moderate-to-vigorous physical activity, and sleep – results in the reduction of time available for other behaviours, given the finite nature of a day (24 h). This method is increasingly used to assess the combined role of movement behaviours for health [7].

In a recent paper published in the International Journal of Behavioural Nutrition and Physical Activity, the authors have conducted a compositional data analysis within the *waking period* (from waking up in the morning to sleep onset) to examine the impact of sedentary behaviour, light physical activity and moderate-to-vigorous physical activity on cardiovascular disease incidence [8]. This choice was made on purpose although data during the sleep window (from sleep onset to last awaking before standing for the day) were available. One of the reviewers required the authors to either include the sleep period within their compositional data analysis or justify their choice. The authors’ primary justification was time awake during the night cannot be seen as an individual choice.

Sleep is a critical part of the 24-h movement behaviour. Including the sleep period requires investigators to classify breaks in sleep period. Some include these breaks as part of the sleep time [7] while others classify it based on the activity levels, [9] although this aspect is not always clearly stated in research articles. However, none of the two options is satisfactory as the first option (sleep break = sleep) assumes that sleep breaks are physiologically similar to sleep, and the second (sleep break = sedentary behaviour/physical activity) equates the role of activity movements during the day to the one during the night. Another option would be to create a separate category defined as “wake after sleep onset” (WASO) or “sleep-related behaviour” as proposed by Gibbs & Kline [3]. This approach might be challenging for compositional data analysis given the potential high number of zeros in the distribution of this measure as previously reported [9]. In populations where WASO are common (and zero values are less problematic), it would be interesting to see the impact of differentiating such behaviour from sleep, sedentary time and physical activity on the findings. Careful attention should be given when interpreting these findings. Results from compositional data analysis are often interpreted in terms of theoretical time reallocation from one movement behaviour to another, even in studies using cross-sectional data, wherein it is more appropriate to compare difference in health outcomes of several time-use compositions. Including WASO as a time-use component of the whole day in such an analysis could help describe the association between WASO and health while accounting for other behaviours. If studies do intend to report results in terms of reallocating time from WASO to other behaviours or vice versa, caution should be given that reallocating 10 min of WASO to sedentary time versus reallocating the same amount to MVPA might be differently associated with health outcomes due to their proportional differences.

We encourage cautious interpretation of the findings from compositional data analysis over a 24-h period, particularly in population where sleep breaks are frequent. Researchers should at least provide information on how time spent awake during the sleep period was classified in the analysis and its distribution (mean, standard deviation) in the study population, to allow the readers to self-assess the potential impact of sleep disturbance on the findings. Investigators are showing interest in this regard. Some have reported WASO in their population [9] and others provided results from compositional data analysis of sleep, sedentary behaviour, and physical activity, while adjusting for sleep characteristics (timing, efficiency, and variability) [10]. This is a first considerable step forward.

## Conclusion

At the time of increasing interest in providing 24-h recommendations for physical activity, sedentary behaviour, and sleep, such as in the recent Canadian guideline [11], it is critical to move forward by building close collaborations between experts in both physical activity and sleep research. Further methodological research is needed to develop holistic approach such as for circadian rhythm, which could encompass information on physical activity, sedentary behaviour, sleep duration and disturbance, while still considering the finite nature of a day. The large amount of data recorded by sensor devices is a great opportunity to develop new, pertinent analytical approaches but this will be only achieved if we combine insights from different fields of expertise.

## Data Availability

Not applicable.

## Abbreviation

WASO: wake after sleep onset
